# Protoporphyrin IX-loaded laminarin nanoparticles for anticancer treatment, their cellular behavior, ROS detection, and animal studies

**DOI:** 10.1186/s11671-019-3138-0

**Published:** 2019-09-18

**Authors:** Yueming Yu, Bingjie Wang, Chunjing Guo, Feng Zhao, Daquan Chen

**Affiliations:** Collaborative Innovation Center of Advanced Drug Delivery System and Biotech Drugs in Universities of Shandong, School of Pharmacy, Yantai University, Yantai, 264005 China

**Keywords:** Laminarin, PDT, Pp IX, Anti-tumor, Nanomedicine, MCF-7 cells

## Abstract

Laminarin conjugate-based nano-scaled particles were in this study proposed as a delivery system for protoporphyrin IX (Pp IX) in photodynamic therapy (PDT) of human breast cancer cells (MCF-7). Hematin-Laminarin-Dithiodipropionic Acid-MGK, named as HLDM, was an amphiphilic carrier material with dual pH/redox sensitive that could be used to load hydrophobic drug to improve their solubility and enhance biocompatibility. Therefore, we combined photosensitizer (Pp IX) with HLDM to fabricate a novel nano-micelles, herein called Pp IX-loaded HLDM micelles. The Pp IX-loaded HLDM micelles were 149.3 ± 35 nm sized in neutral water. Phototoxicity, in vitro PDT effect, and dual sensibility to pH and redox microenvironment of Pp IX-loaded HLDM micelles were examined at different concentrations by using MCF-7 human breast cancer cells. The experiments on phototoxicity and reactive oxygen species (ROS) production proved that the micelles could produce PDT to kill the cancer cells with a certain wavelength light. The apoptosis experiment indicated that the micelles could cause nuclear damage. In vivo PDT effect of the micelles was studied by constructing the tumor-bearing nude mouse model of MCF-7 cells. In vivo studies showed that the Pp IX-loaded HLDM micelles could induce remarkable anti-tumor effect. A promising laminarin-based nanomedicine platform acts as a new drug delivery system to enhance the uptake, accumulation, and PDT efficacy of Pp IX in vitro and in vivo.

## Introduction

Photodynamic therapy (PDT) [1–3] is an established type of therapy affected by light source, photosensitizer, and molecular oxygen [4], which can produce reactive oxygen species (ROS)-mediated [5, 6] direct lethal effects on cancer cells within the illuminated area under the condition of minimally invasive nature [6] and low toxicity. It can give rise to DNA damage [7], activate signaling pathways to facilitate vasculotoxic reaction that interdicts blood supply to the tumor area [8], and provoke tumor cells recognition and destruction by the immune system [9]. The ultimate effect is overcoming drug-resistance [10, 11] and eliciting a selective antitumor effect, resulting in cancer cell death.

At present, traditional treatments for tumor [12], such as radiotherapy, chemotherapy, and surgery, are widely used in clinic, but these methods have great toxic and side effects, great trauma, great risk, certain limitations, and easy recurrence. PDT is used in the treatment of extensive cancer, including breast [13–15], hepatocyte [14], lung [16], melanoma [17], and skin [18] cancer, becoming the focus of domestic and foreign researchers. Experience has proved that PDT is a better choice to alternate conventional methods like chemotherapy [19] and surgery in the therapy of various diseases and tumors [20], as it has advantages such as inhibiting cancer metastasis [21] and being selective and adaptable. However, the applications of most photosensitizers have been limited in cancer therapy because of their limited accumulation at the tumor site [22].

Protoporphyrin IX (Pp IX) is a hydrophobic photosensitizer [23, 24] that has great potential for use in diagnosis and PDT. Pp IX is a hematoporphyrin derivative that can also elicit apoptosis without external stimulus (such as laser light [25]), manifesting that it is likely to have novelty function to remedy the cancer [26].

Topical accumulation of Pp IX in premalignant and malignant lesions is thus an interesting strategy to be furnished [27], as its fluorescence provides a convenient method for tumor orientation [28].

However, Pp IX has some disadvantages that need to be solved [29], such as it has poor solubility and is easy to aggregate in aqueous solution, resulting in a reduced efficacy. Therefore, laminarin [30] is a marine nanomedicine carrier biomaterial that is used as hydrophilic group carrier to improve the unfavorable features of photosensitizers. It has been proven that laminarin owns effective biological activities, including antitumor, antiviral, and so on. Accumulating evidence suggests [31] that it has good therapeutic efficiency on different types of cancer cells in vitro and in vivo (such as breast and colon cancer cells [32]).

Stimulation-responsive polymer nanoparticles, such as liposome and micelles, can further ensure drug delivery and reduce side effects. Liposomes [33] can be used as diagnostic and therapeutic tools, and amphotericin B can be incorporated into liposomes to treat the fungal infections [34]. Polymeric micellar nanoparticles [35] are a smart drug delivery [36]. The Pp IX-loaded Hematin-Laminarin-Dithiodipropionic acid-MGK (HLDM) micelles with dual pH/redox sensitive and photoresponse contain a hydrophobic core for loading Pp IX as well as a laminarin hydrophilic shell. They have been one of the most significance nanoscopic drug delivery to improve the unfavorable features of photosensitizers [37], such as drug biodistribution, adverse effects, and drug-loaded release [38, 39].

Regarding this, we therefore designed a multifunctional drug delivery nanoplatform [40] based on laminarin in response to pH [41] and redox properties [42], which could maintain the solubility in water and quench the Pp IX in a human body’s blood circulation while dequenching the Pp IX in targeted sites [43]. Internal and external stimuli responsive type of drug delivery has received extensive attention, such as temperature [44], ultrasound [45], pH, and redox. A thermoresponsive system has been studied to control drug delivery, showing potential for better drug delivery [46]. The stimuli-responsive drug delivery system has promoted continuous drug on-demand release [47] irreversibly and distributed rapidly.

**Fig. 1:**
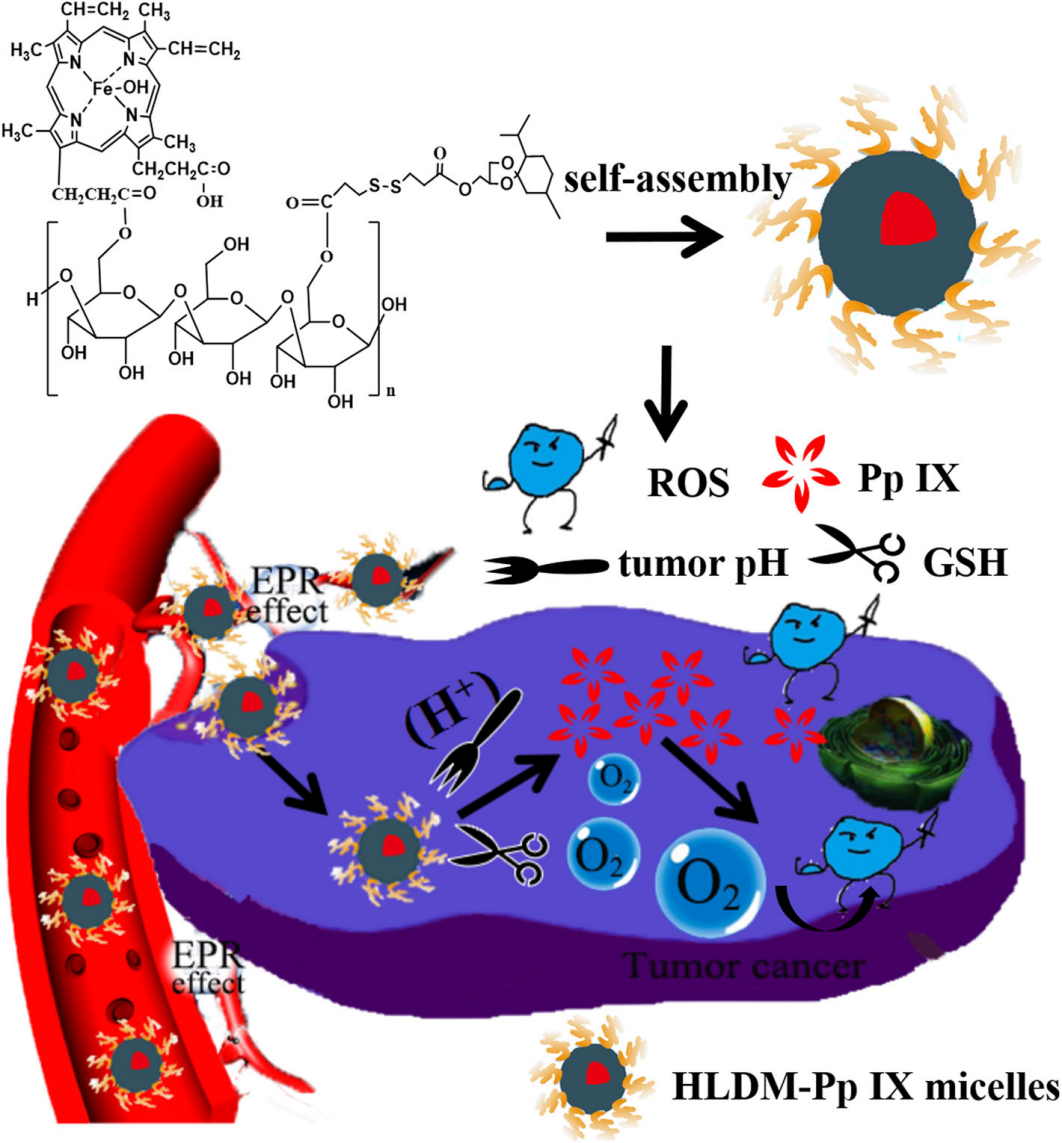
Schematic illustration of laminarin-based nanomedicine (HLDM) used to deliver the photosensitizer for tumor therapy

In this study, Pp IX-loaded HLDM micelles were prepared to overcome some disadvantages of Pp IX [48], such as instability and aggregation in the aqueous solution. We hypothesized that the Pp IX-loaded HLDM micelles, self-assembled from HLDM nanocarriers [49], should be accumulated and released Pp IX in the tumor microenvironment [50]. Pp IX was stimulated by to facilitate ROS generation after the accumulation of Pp IX in tumor cells, which might cause cancer cell death (as in Fig. 1). The synthesis and characterization of HLDM materials had been proved by 1H-NMR as previously reported [51]. So, in the present work, the cellular uptake, phototoxicity, ROS generation, nuclear morphological observation, and in vivo PDT effect of Pp IX-loaded HLDM micelles were studied.

## Methods

### Materials

Laminarin was purchased from Sigma-Aldrich (Shanghai, PR China). Dimethyl sulfoxide (DMSO) was supplied by Tianjin Bodi Co. Ltd. l-glutathione (GSH), Hoechst 33342 were provided by Sigma-Aldrich (Shanghai, PR China). Dulbecco’s modified Eagle’s medium (DMEM) and fetal bovine serum (FBS) were obtained from Science Biotechnology Co. Ltd. (Shangdong, Yantai, China). Reactive oxygen species (ROS) assay kit was provided by Beyotime Biotechnology (Shanghai, China). H*&*E were purchased from Bioworld Technology Co. Ltd. (Nanjing, China). Pp IX was supplied by Aladdin Reagent Net (Shanghai, China). All other reagents and solvents were of chemical grade.

Human breast cancer cells (MCF-7) were supplied by the Laboratory of Molecular Pharmacology, at the School of Pharmacy of Yantai University (Shandong, China).

Female nude mice weighing 14–18 g (3–4 weeks) were purchased from Beijing Vital River Laboratory Animal Technology Co. Ltd.

### Synthesis and Characterization of HLDM Materials

The HLDM materials were synthesized and provided by using the methods as presented in previous reports [51]. Firstly, oxaloyl chloride was used to activate dithiopropionic acid into acyl chloride, which was acylated with MGK to obtain HOOC-S-S-MGK. After that, 1-ethyl-3-(3-dimethylaminopropyl) carbodiimide hydrochloride (EDCI) and 4-dimethylaminopyridine (DMAP) were used to activate HOOC-S-S-MGK, and then esterification reaction was carried out with laminarin at 40°C. Finally, we synthesized the HLDM materials by esterification using EDC/DMAP as catalyst. The DMSO-D_6_ and D_2_O were chosen as solvent to analyze the composition of the compounds. And ^1^H-NMR (Advance Bruker 400M; Switzerland Bruker Company, Madison, WI, USA) spectra, IR spectra, and UV-visible absorption spectra (200–700 nm) for HLDM materials were tested and determined at room temperature.

### Preparation of Self-Assembly Micelles (Pp IX-Loaded HLDM Micelles)

The Pp IX-loaded HLDM micelles were exploited via dialysis method. In a nutshell, a hydrophobic core consists of MGK, dithiodipropionic acid, and hematin as well as a laminarin hydrophilic shell could self-assemble in water to form polymicelles. Pp IX was loaded in the hydrophobic core during agitation to gain Pp IX-loaded HLDM micelles. HLDM and Pp IX were dialyzed in deionized water (MWCO 2000 Da) on 90-1 stirrer at 600 rpm after stirring for reasonable time in organic reagent to dissolve, followed by subsequent processing, to gain Pp IX-loaded HLDM micelles. The whole procedure took place at room temperature.

### Characterization of Micelles

Particle size and zeta-potential for the Pp IX-loaded HLDM micelles were determined by using Beckman Coulter Particle Analyzer (part number:A35878) at room temperature. The morphology of the Pp IX-loaded HLDM micelles was visualized by a H-600 transmission electron microscope (H-600 TEM; Hitachi, Tokyo, Japan). To determine loading capability, Pp IX-loaded HLDM micelles were broken by an ultrasonic apparatus in an organic reagent. The concentration of free Pp IX in micelles was measured by UV-visible absorption spectra at 630 nm. The entrapment efficiency (EE) and drug loading content (DL) were calculated according to the formula.

EE (%) = (weight of Pp IX in the Pp IX-loaded HLDM micelles/weight of the overall Pp IX) ×100%

DL (%) = (weight of Pp IX in the Pp IX-loaded HLDM micelles/weight of the micelles) ×100%

### Cell Culture

The human breast cancer cell lines (MCF-7), colon cancer cell lines (CT-26) (Fig. 5), and lung cancer cell lines (A549) (Fig. 5) were used to determine Pp IX-loaded HLDM micelles by inverted fluorescence microscope (AxioVert.A1). It had been broadly proved preliminarily that these materials had anti-tumor effect. But the experiment demonstrated that MCF-7 could have more uptake than the other two cancer cell lines. Therefore, MCF-7, cultured in DMEM (Hyclone) with 10% fetal bovine serum, was selected to monitor the curative effect at 37 °C in a humidified atmosphere containing 5% CO_2_.

### Cell Uptake

The fresh medium containing free Pp IX, Pp IX-loaded laminarin-hematin (LH) micelles, or Pp IX-loaded HLDM micelles were added to replace the original medium after 24 h, respectively. The MCF-7 cells were then cultured for 1 h, 2 h, and 4 h (Pp IX concentration: 20 μg/mL) or for 4 h with following different concentrations of Pp IX :10 μg/mL, 20 μg/mL, and 50 μg/mL in above atmosphere. The consequence of cellular uptake was observed by inverted fluorescence microscope (Eclipse E400; Nikon Corporation, Tokyo, Japan) to have a qualitative analysis [52].

### Cell Location Study

In this study, Pp IX was not only an anticancer drug to elicit cancer cell death but also a red fluorescence probe to locate the uptake. The MCF-7 cells in fresh medium containing free Pp IX, Pp IX-loaded LH micelles, or Pp IX-loaded HLDM micelles were cultured for 4 h at 20 μg/mL concentration above atmosphere. After fixing with 4% paraformaldehyde, the fixative was replaced by Hoechst 33342 (10 μg/mL) to dye the nucleus for 15 min. The result for location was visualized by inverted fluorescence microscope.

### Measurement of Reactive Oxygen Species Generation

The generation capability for reactive oxygen species (ROS) was measured intracellularly using fluorescence microscope, which used the ROS probe 2′,7′-dichlorofluorescin diacetate (DCFH-DA). MCF-7 was seeded into six-well plates and incubated. After 24 h, the medium was removed and replaced with fresh medium containing free Pp IX or Pp IX-loaded HLDM micelles (20 μg/mL) for 2 h. The cells were washed with DMEM medium, followed by half an hour irradiation (630 nm). After washing twice, MCF-7 cells were incubated with DCFH-DA (10 μmol/L) at above atmosphere for 30 min, which were then imaged by fluorescence microscope (excitation wavelength: 488 nm, emission wavelength: 525 nm) after washing again with DMEM medium.

### Phototoxicity and Viability Assays

MCF-7 was inoculated in for 96-well plant to detect the cytotoxicity of different dosage forms for the viability assays. Then the fresh DMEM including different concentrations of free Pp IX, Pp IX-loaded LH micelles, or Pp IX-loaded HLDM micelles (1, 2, 5, and 10 μg/mL) were added in each well. For the group of phototoxicity, the cells were incubated for 4 h to absorb, and they were further irradiated for 30 min, followed by incubation for 24 h at above atmosphere. On the other hand, the wells were set up to analyze the cytotoxicity and viability in dark condition as control group. They were further inoculated for 24 h at above atmosphere.

Twenty microliters of 3-(4,5-dimethylthiazole-2-yl)-2,5-diphenyl-tetrazolium bromide (MTT) solution (5 mg/mL) and 180 μL PBS (pH 7.4) were then added in 96-well plate and further incubated for another 3 h. Subsequently, 150 μL DMSO was used to solute the formazan product and the absorbance (OD) was measured using enzyme-labeled instrument (SpectraMax M 5) at 490 nm. The MCF-7 viability was expressed by using the following formula:

Viability = ((OD sample-OD black)/(OD control-OD black))×100%.

The values of OD sample were provided by the drug treated cells, while the values of OD control were supplied by the cells without drug, and the values of OD black were obtained from the wells without drug and cells.

### Nuclear Morphological Observation

MCF-7 cell line was incubated for 24 h, and then stimulated with Pp IX-loaded HLDM micelles for 4 h. After rinsing and fixation, the cells were stained with a nuclear fluorescence probe for 20 min at 37 °C, followed by removal of the dye from the environment using PBS. The corresponding fluorescence images were visualized using a fluorescence microscopy.

### In Vivo Efficacy and Safety Evaluation

Female nude mice were afterwards used to investigate the anticancer feasibility of Pp IX-loaded HLDM micelles in vivo. MCF-7 cells (1.5 × 10^6^ cells/0.1 mL) were injected in oxter of female nude mice as animal models, and then estrogen was given by intragastric gavage to promote tumor growth. The mice had been randomly divided into five groups once the volume of tumors reached approximately 70–100 mm^3^, which were denoted as normal saline, free Pp IX (5 mg/kg), Pp IX-loaded HLDM micelles (5 mg/kg of free Pp IX equivalents), free Pp IX (5 mg/kg) plus light irradiation, and Pp IX-loaded HLDM micelles (5 mg/kg of free Pp IX equivalents) plus light irradiation. The light-treated groups were exposed in 630 nm laser with 30 min at 24 h post-injection. The therapeutic efficacy was evaluated by monitoring the tumor volumes in five treated groups every other day and analyzing the histopathological slide after 20 days. And body weights were measured to appraise the drug safety in five treated groups every 2 day [53].

### Statistical Analysis

All data in this study were recorded as Means ± standard deviation (*n* = 3). Moreover, significant differences between different groups were analyzed using one-way analysis of variation (ANOVA). The differences were deemed to be statistically significant at probability levels of *P < 0.05 (significant), **P < 0.01 (highly significant).

## Results and Discussion

### Characterization of HLDM materials

**Fig. 2:**
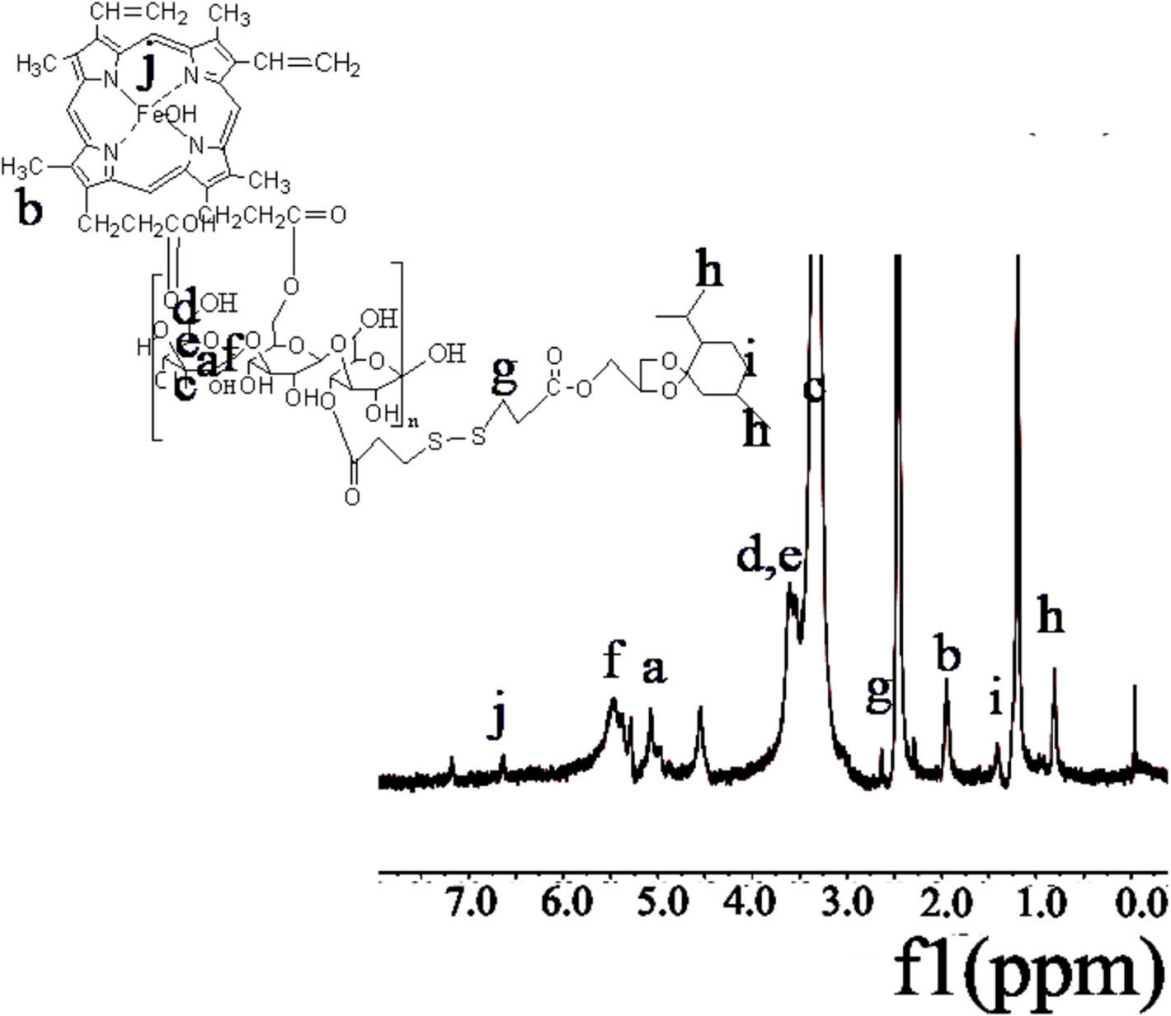
The ^1^H-NMR spectra of HLDM

^1^H-NMR spectra for the HLDM materials were shown in previous reports [51]. The methyl peak for MGK was observed at about δ: 0.8 (Fig. 2h). ^1^H-NMR spectra revealed absorption peak at about δ: 2.8 (Fig. 2g), which was CH_2_ in 3,3-dithiodipropionic acid. The appearance of the signal peak at δ: 6.5 (Fig. 2j) verified the presence of hematin. The characteristic peak for laminarin in amphiphilic polymer materials was found in the region between 3 and 4 ppm, indicating that the new product of HLDM had been synthesized successfully.

### IR Spectra for HLDM

IR spectra of HLDM materials were shown in previous reports [51]. The double-peak in the picture testified the connection of the MCK. Moreover, the characterized peak of ester carbonyl group was observed in IR spectra.

### UV-Visible Absorption Spectra of HLDM

**Fig. 3.**
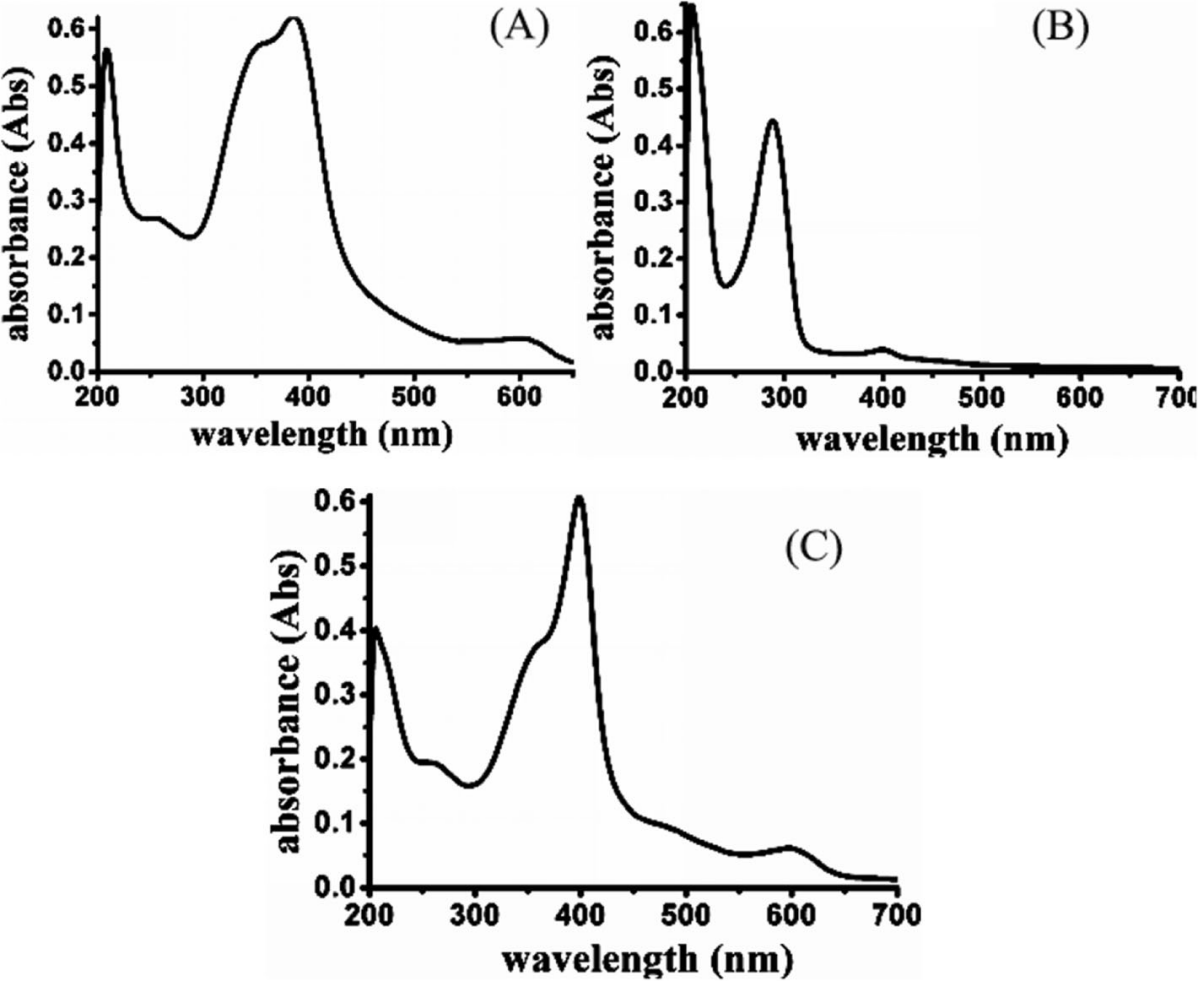
UV-visible absorption spectra of hematin (**a**), Laminarin-S-S-MGK (**b**), and HLDM (**c**)

In Fig. 3a, hematin had an ultraviolet absorption wavelength (about 580 nm) and in Fig. 3b, Laminarin-Dithiodipropionic acid-MGK had no absorption in the same position. The UV-visible absorption spectra were accomplished to verify the link of hematin based on this. The outcome indicated that the characterized absorption wavelength in 580 nm was observed in HLDM materials (Fig. 3c). Hematin had been connected successfully to HLDM materials.

### Characterization of Pp IX-Loaded Micelles

**Fig. 4:**
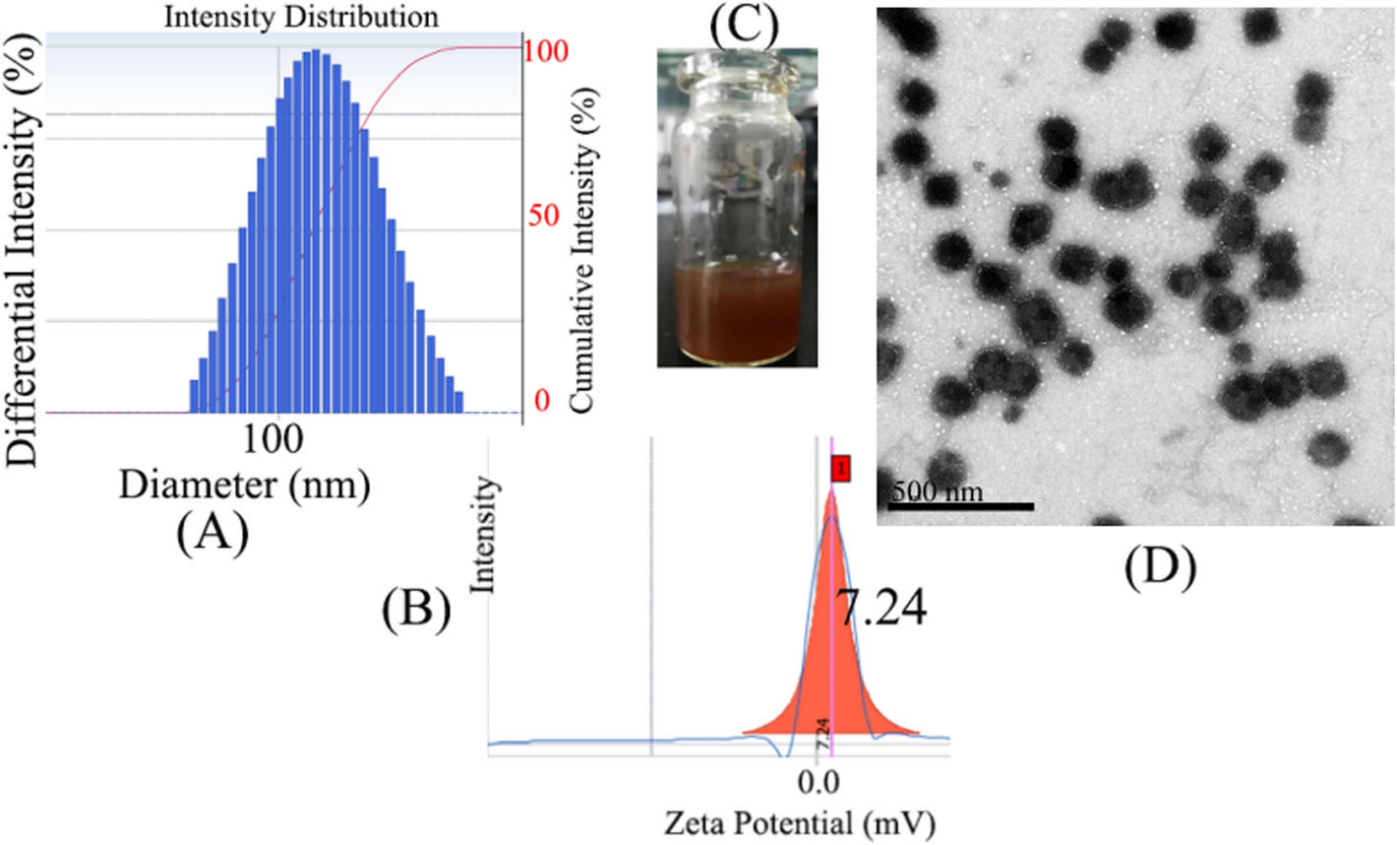
**a**, **b** The size and zeta potential of Pp IX-loaded HLDM micelles. **c** The Pp IX-loaded HLDM micelles in water. **d** The TEM image of Pp IX-loaded HLDM micelles

**Fig. 5:**
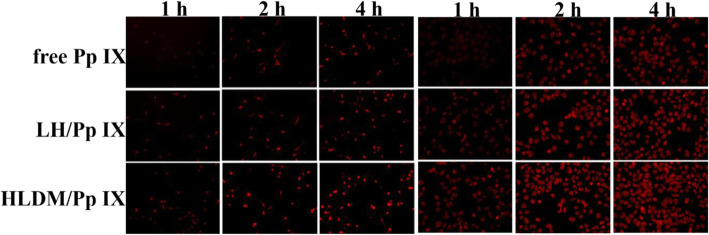
Uptake of free Pp IX, Pp IX-loaded LH, and Pp IX-loaded HLDM micelles in CT-26 (left) and A549 (right)

The size and zeta potential of Pp IX-loaded HLDM micelles are shown in Fig. 4a, b. It was shown that the micelles had been absorbed better by the cancer cells, to enhance efficiency and reduce side effects (enhanced permeability and retention effect, EPR). The micelles were seen by naked-eye after Millipore membrane filter in Fig. 4c. On this basis, the picture of Pp IX-loaded HLDM micelles was scanned by transmission electron microscope (TEM) as shown in Fig. 4d. The morphology was nonuniform particles, which was because the time for ultrasound was deficient. On the other hand, the agglomeration of particles was observed in the picture, probably because of the higher concentration (Fig. 5).

The entrapment efficiency (EE) and drug loading content (DL) were calculated by the formula (Table 1). It was discovered after many experiments that the fluctuation of EE and DL were unstable, as we had speculated that the Pp IX-loaded HLDM micelles might aggregate in aqueous solution.

**Table 1:** The physiochemical properties of different micelles (*n* = 3)

Group	Size (nm)	ZP (mv)	DL (%)	EE (%)
Pp IX-loaded LH micelles	181.2 ± 10	13.77 ± 5.36	4.61 ± 4.3	45.64 ± 6.24
Pp IX-loaded HLDM micelles	149.3 ± 35	7.24 ± 4.9	5.89 ± 3.87	40.06 ± 4.58

### Cellular Uptake

**Fig. 6:**
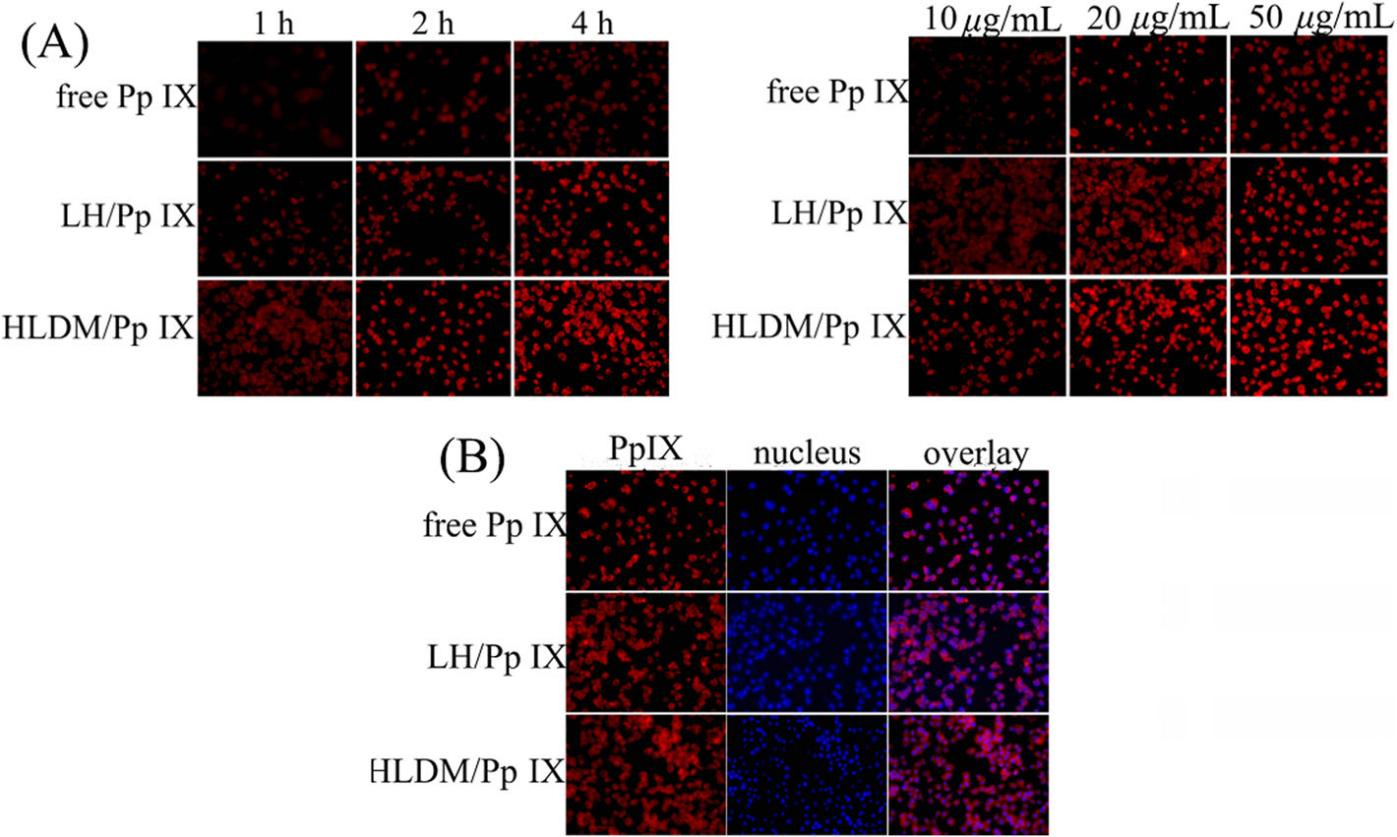
**a** Uptake of free Pp IX, Pp IX-loaded LH, and Pp IX-loaded HLDM micelles. **b** Cell location of Pp IX-loaded HLDM micelles

In this study, the fluorescence of Pp IX was detected to investigate the time and concentration dependence. As seen from the diagram, the Pp IX-loaded HLDM micelles were absorbed in MCF-7 cells and their fluorescence intensity increased with time and concentration. By comparing the three micelles in Fig. 6a, the cancer cells that were given Pp IX-loaded HLDM micelles had more fluorescence. This was because pH/redox moieties had been linked to the materials to respond to the tumor microenvironment. The cancer cells that were given free Pp IX had weaker fluorescence because of the aggregation in DMEM.

From what has been discussed above, we may safely draw the conclusion that HLDM materials, including pH and reduction sensitivity moieties, can improve the agglomeration of Pp IX and enhance their absorption and release in tumor cells.

### Cell Location Study

As shown in Fig. 6b, the nucleus was stained by fluorescent dye, and then we could see the phenomenon red fluorescence presented outside of the blue fluorescence. We had speculated that the cell uptake might be related to cytoplasm, so this hypothesis was verified by the previous study that Pp IX had been accumulated and localized in the mitochondria and cytoplasm of tumor cells [54].

### Measurement of Reactive Oxygen Species Generation

**Fig. 7:**
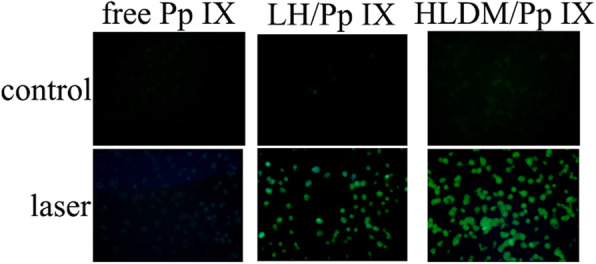
Reactive oxygen species (ROS) generation under light condition

As shown in Fig. 7, the reactive oxygen species (ROS) in MCF-7 cells was monitored by using DCFH-DA as an indicator, which had been observed to have green fluorescence in fluorescence microscopy. Pp IX-loaded HLDM micelles had stronger intensity of green fluorescence under the light while free Pp IX had hardly fluorescence. We had speculated that free Pp IX might agglomerate to cause self-quenching effect in DMEM. The green fluorescence of three groups was negligible without light (such as control group). These results confirmed that Pp IX could stimulate oxygen to generate ROS as a photosensitizer under the condition of light.

### Phototoxicity and Viability Assay

**Fig. 8:**
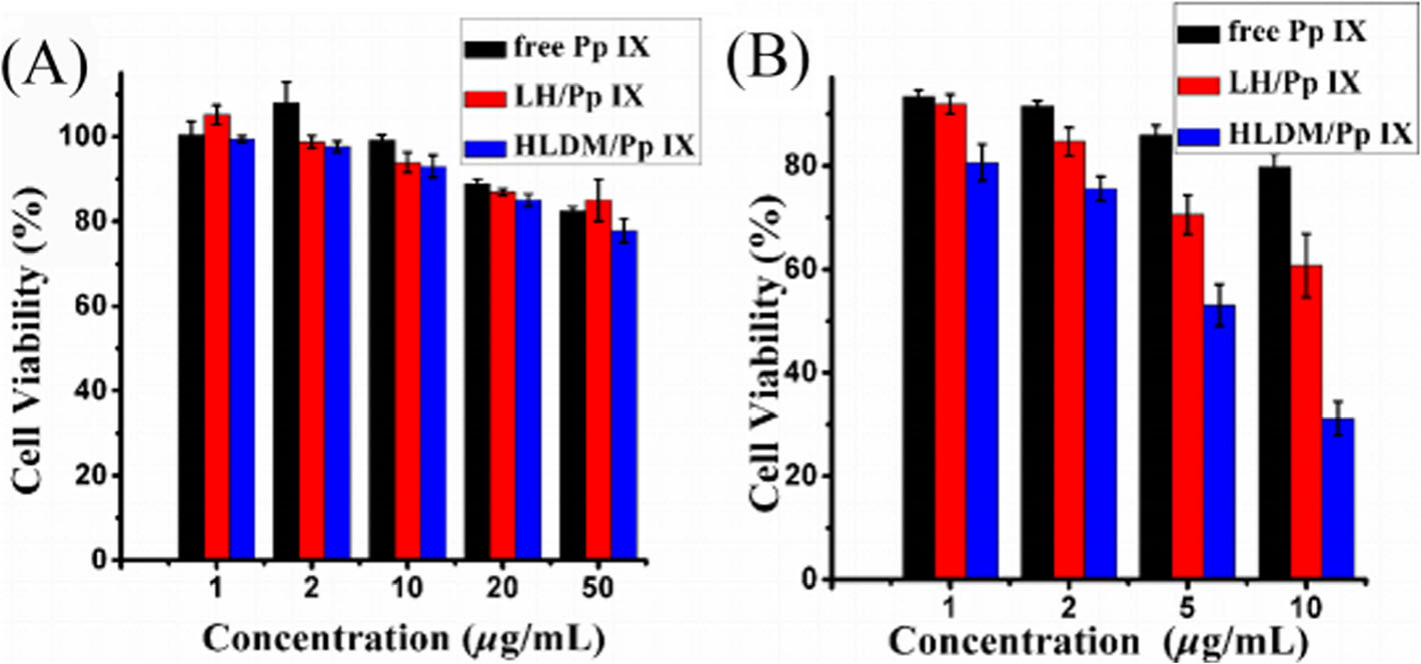
**a** MCF-7 cells viability of free Pp IX, Pp IX-loaded LH micelles, or Pp IX-loaded HLDM micelles under light condition. **b** Relative light-toxicity of free Pp IX, Pp IX-loaded LH micelles, or Pp IX-loaded HLDM micelles upon irradiation. *n* = 3; * indicates *P <* 0.05

Cell cytotoxicity and viability assay were performed with the human breast MCF-7 cancer cells under two different external environments, using the MTT assay. As shown in Fig. 8a, the significant cell damage difference was negligible in all samples under darkness. When the Pp IX concentration was increased to 50 μg/mL, the MCF-7 cells viability that we detected remained at high level. The phenomenon showed that the cytotoxicity to cells or organs was not augmented significantly with increased concentration of Pp IX.

As depicted in Fig. 8b, *5* μg/mL of Pp IX had significant difference in free drugs and micelles groups. The cytotoxicity to cells or organs was augmented significantly in micelles group, as the concentration of Pp IX increased under the light, while the free Pp IX groups showed little change up to a concentration of 10 μg/mL. These data showed that the phototoxic efficiency of Pp IX-loaded micelles was clearly higher than that of free Pp IX. Once again, the experiment demonstrated that free photosensitizer could accumulate to cause self-quenching effect. Therefore, we can conclude that the Pp IX-loaded HLDM micelles have a huge potential for killing cancer cells with light irradiation.

### Nuclear Morphological Observation

**Fig. 9:**
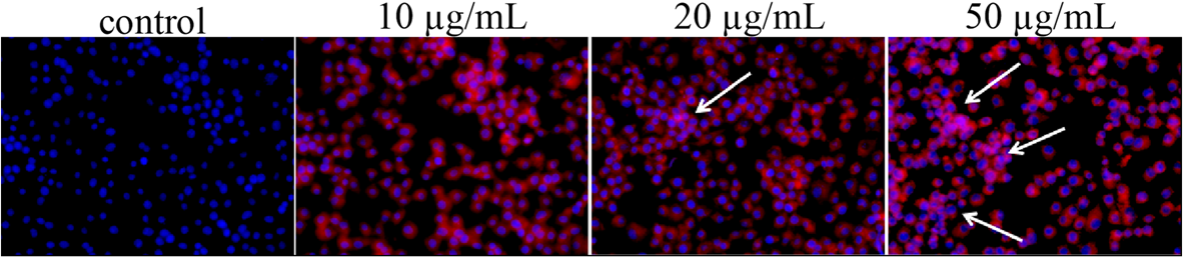
DNA damage of MCF-7 cells after Pp IX treatment

In cell location study, we discovered unwittingly that the stained nucleus showed white spots, and the higher concentration of Pp IX was more obvious with this phenomenon. Maybe this was because of DNA damage in nucleus. As shown in Fig. 8, 20 μg/mL Pp IX-loaded HLDM micelles could cause DNA damage compared to the corresponding control in MCF-7. When the concentration reached 50 μg/mL, the damage would be serious in cancer cells. The nuclear morphological observation study suggested that DNA damage was an early marker for MCF-7 cells death that was induced by Pp IX [26] (Fig. 9).

### In Vivo Efficacy and Safety Evaluation

**Fig. 10:**
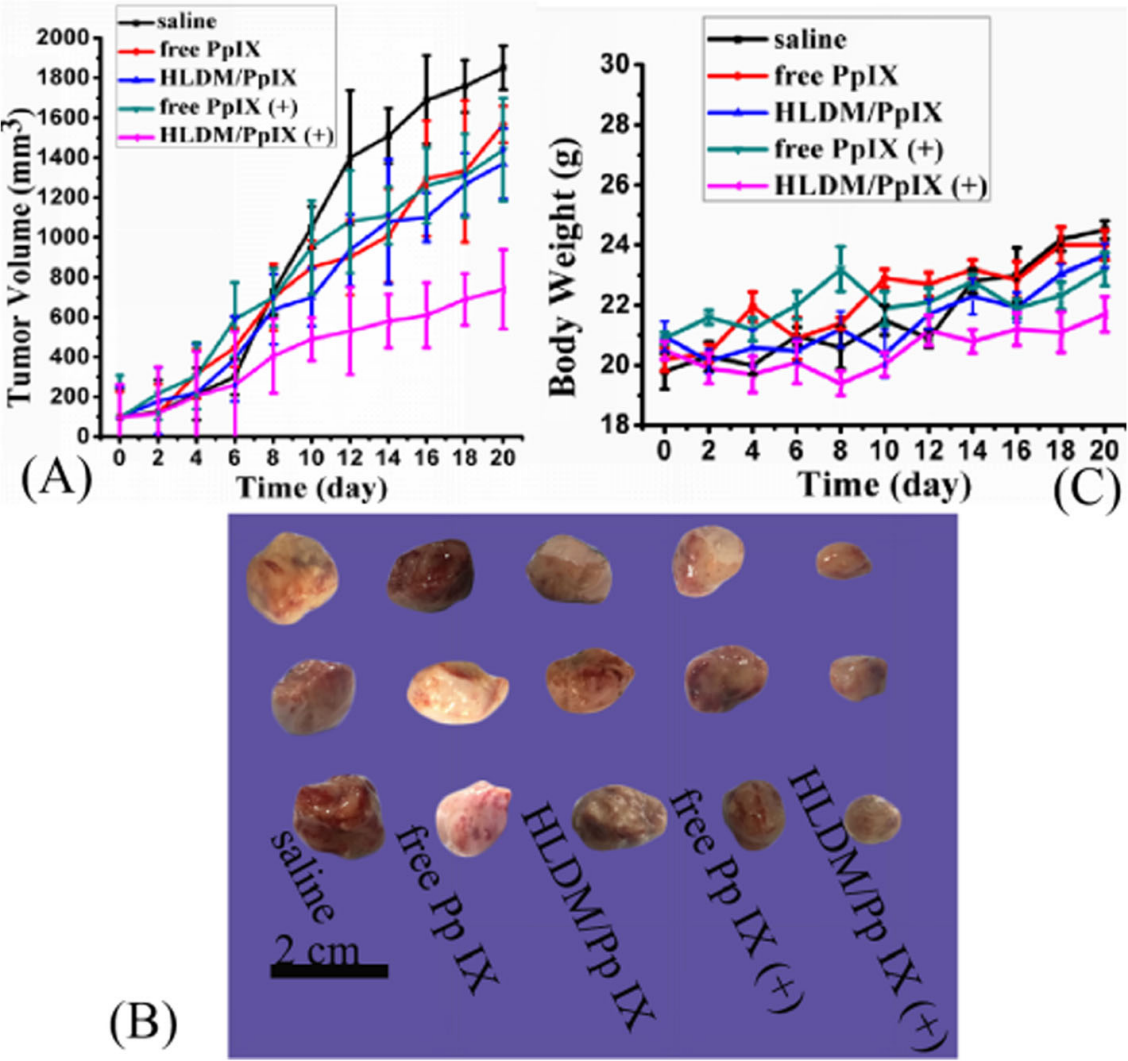
In vivo antitumor activity and safety evaluation. **a** Tumor volume changes over treatment times. **b** Tumor volume of five groups: (**a**) normal saline, (**b**) free Pp IX (5 mg/kg), (**c**) Pp IX-loaded HLDM micelles (5 mg/kg of free Pp IX equivalents), (**d**) free Pp IX (5 mg/kg) plus light irradiation, and (**e**) Pp IX-loaded HLDM micelles (5 mg/kg of free Pp IX equivalents) plus light irradiation. **c** The body change of tumor-bearing nude mice

As shown in Fig. 10a, b, the tumor growth of five groups was measured to evaluate efficacy in vivo. The group treated with saline exhibited continuous growth at a relatively high rate. There was no significant difference between the groups treated with free Pp IX and Pp IX-loaded HLDM micelles and the saline group. These data indicated that the tumor volume was less affected by Pp IX without irradiation. Meanwhile, the group treated with free Pp IX plus light produced slight change in tumor volume. What caused this phenomenon was that the free drug was unstable in vivo and was thus easy to collect in blood. Therefore, it might have been eliminated before getting to the tumor tissue. In contrast, the tumor growth treated with Pp IX-loaded HLDM micelles was significantly inhibited in Fig. 10a. This phenomenon proved that the micelles exhibited significant antitumor effect after giving a certain wavelength of light to stimulate. To sum up, this experiment demonstrated that the antitumor effects of Pp IX-loaded HLDM micelles had improved obviously under the light condition.

On the other hand, the relative body weight was measured to evaluate the safety of Pp IX-loaded HLDM micelles (Fig. 10c). There was no evident body weight loss and negligible changes in all the groups, suggesting the good biosafety of these treatments to the mice.

**Fig. 11:**
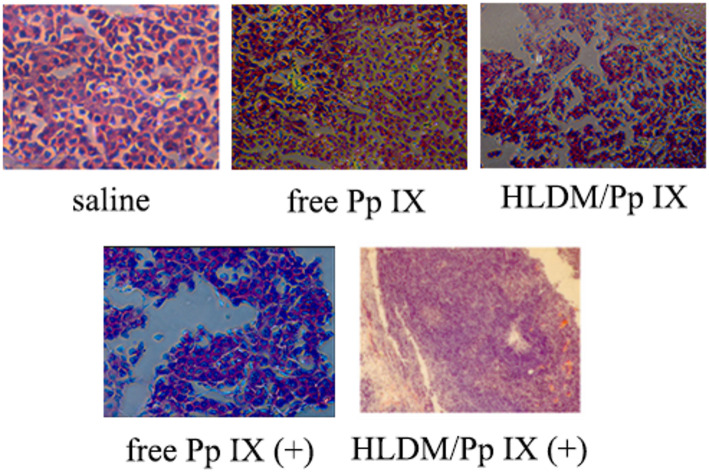
H&E tumor staining with different formulations. All data are reported as a mean ± SD. *n* = 3; * indicates *P <* 0.05

Furthermore, the histopathologic slide showed a clear nuclear polymorphism in the saline group in Fig. 11. The pathological changes in the tumor tissue stained with hematoxylin and eosin (H&E) had significant difference in five groups. The results showed a slight nuclear condensation in the Pp IX-loaded HLDM micelles and free Pp IX groups. The tumor tissues from the Pp IX-loaded HLDM micelles (plus light) group exhibited an obvious nuclear damage. Hence, we concluded that these results were consistent with the above results for in vivo efficacy and safety evaluation.

To date, a variety of materials have been studied for drug delivery [55]. In the previous study, we successfully synthesized dual pH/redox sensitive [56] marine polysaccharide laminarin conjugates, and in this research, the conjugates were used as delivery system for Pp IX, to achieve anti-tumor effects. In vivo experiments manifested that Pp IX-loaded HLDM micelles could effectually deliver Pp IX into cancer cells and generate ROS-mediated direct lethal effects on cancer cells. The cytotoxicity experiments showed that the micelles had slight cytotoxicity without light irradiation, while low concentration solutions of micelles had a noticeable impact on cell viability within a certain illumination. In animal level, Pp IX-loaded HLDM micelles exerted phototoxic effect to produce a relevant anti-tumor effect. Therefore, the activities of Pp IX-loaded HLDM micelles were convincingly certified in vitro and in vivo.

## Conclusions

A novel laminarin-based nanomedicine platform to address undesirable characteristics of Pp IX such as instability and astatic distribution was successfully studied in this research. The photosensitivity and phototoxicity of Pp IX-loaded HLDM micelles were detected and evaluated in vitro and in vivo. Nuclear morphological observation of Pp IX showed that the Pp IX-loaded HLDM micelles could effectively deliver and accumulate Pp IX to cancer cells and cause nuclear damage. The research on phototoxicity and ROS production manifested that Pp IX-loaded HLDM micelles exhibited a relevant PDT effect, exerting anti-tumor activity with a certain wavelength light. Likewise, the in vivo research testified that the Pp IX-loaded HLDM micelles could induce PDT effect under the light condition, which could remarkably enhance the anti-tumor effect of Pp IX. To sum up, the results for in vitro and in vivo studies indicated that Pp IX-loaded HLDM micelles could effectively produce PDT effect and can be applied in the future in tumor treatment in the next research. This promising laminarin-based nanomedicine platform will have great potential for becoming new drug delivery system [57] to deliver hydrophobic photosensitizer for cancer photodynamic therapy (PDT).

## Data Availability

The datasets supporting the conclusions of this article are included within the article.

## Abbreviations

HLDM: Hematin-Laminarin-Dithiodipropionic acid-MGK
LH: Laminarin-Hematin
Pp IX: Protoporphyrin IX
PDT: Photodynamic therapy
ROS: Reactive oxygen species
